# SARS-CoV-2 Polymerase Chain Reaction Positivity Rates Among Evacuees From Afghanistan After Withdrawal of the Coalition Forces

**DOI:** 10.1001/jamanetworkopen.2022.13467

**Published:** 2022-05-23

**Authors:** Adeel A. Butt, Naseer A. Masoodi, Arslan T. Jawad, Fatima Haider, Nadya Al Rauili, Salih Al-Marri, Abdul-Badi Abou-Samra

**Affiliations:** 1 Hamad Medical Corporation, Doha, Qatar; 2 Departments of Medicine and Population Health Sciences, Weil Cornell Medical College, New York, New York; 3 Departments of Medicine and Population Health Sciences, Weil Cornell Medical College, Doha, Qatar; 4 Ministry of Public Health, Qatar

## Abstract

This cross-sectional study examines the rate of active SARS-CoV-2 infection among Afghan refugees arriving in Qatar after the withdrawal of coalition forces from Afghanistan.

## Introduction

Afghanistan has been a major armed conflict zone for over 2 decades. Consequently, the response to multiple infectious disease threats, including the SARS-CoV-2 pandemic, has been poor because of a lack of resources and inadequate infrastructure.^1,2^ In August 2021, the Western coalition forces completely withdrew from Afghanistan, which further exacerbated the shortage of critical medicines and supplies.^3^ Qatar was the first stop for a large proportion of evacuees en route to their final destination. We conducted a cross-sectional study to determine the rate of active SARS-CoV-2 infection among evacuees arriving in Qatar from Afghanistan.

## Methods

All evacuees from Afghanistan arrived in Qatar on charter flights through a separate noncommercial airport. Most were quickly transported to their next destination without entering Qatar. Those who entered Qatar between August 20 and December 15, 2021, were subject to Qatar’s routine testing policies and were included in this study.^4^ Each evacuee who entered Qatar was tested for SARS-CoV-2 within 12 hours of entering Qatar at a single reference laboratory using TaqPath COVID-19 Combo Kits (Thermo Fisher Scientific).^4,5^ Age, sex, and nationality were determined by the individual’s passport used for entry. This report complied with the Strengthening the Reporting of Observational Studies in Epidemiology (STROBE) reporting guideline. The study was approved by the institutional review board at Hamad Medical Corporation with a waiver of informed consent requirement because data were collected as a part of the national public health response. Data were analyzed using Excel for Microsoft 365 (Microsoft Corp).

## Results

Between August 20, 2021, and December 15, 2021, 7834 evacuees (median [IQR] age, 23 [11-35] years; 4772 male individuals [54.5%]) entered Qatar and were tested for SARS-CoV-2 infection (7576 individuals [96.7%] were tested within 24 hours). Their demographic characteristics are provided in Table 1. At presentation, 122 individuals (1.6%) reported having upper respiratory infection–like symptoms. Sixteen individuals (0.2%) had a positive or reactive test, with 7 of 16 persons (43.8%) having a cycle threshold value of less than 30. Clinical details of the 16 positive cases are provided in Table 2. Information on the presence of symptoms was available for 13 of 16 persons, with 3 of 13 being symptomatic. Two of those persons had a cycle threshold value of greater than 30.

**Table 1.** Demographics of the Tested Persons

Characteristic	Individuals, No. (%) (N = 7834)
Age group, y	
0-10	1928 (24.6)
>10-20	1571 (20.1)
>20-30	1583 (20.2)
>30-40	1419 (18.1)
>40-50	704 (9.0)
>50-60	359 (4.6)
>60-70	178 (2.3)
>70-80	71 (0.9)
>80-90	21 (0.4)
Sex	
Male	4772 (54.5)
Female	3359 (42.8)
Unknown	209 (2.7)
Nationality	
Afghanistan	5929 (75.7)
Australia	41 (0.5)
Canada	157 (2.0)
European Union	740 (9.4)
India	220 (2.8)
Nepal	44 (0.6)
Pakistan	47 (0.6)
Qatar	52 (0.7)
United Kingdom	159 (2.0)
US	246 (3.1)
Others^a^	199 (2.5)
SARS-CoV-2 reverse-transcriptase polymerase chain reaction	
Positive (cycle threshold value ≤30)	7 (0.09)
Reactive (cycle threshold value >30)	9 (0.12)
Negative	7818 (99.79)

^a^ Others includes 52 other nationalities. The top 10 nationalities in terms of numbers are listed in the table.

**Table 2.** Clinical Details of the Persons With a Positive or Reactive SARS-CoV-2 Polymerase Chain Reaction

Patient No.	Sex and age group, y	Nationality	Date of arrival	Date tested	Symptomatic	Cycle threshold value	Vaccinated
1	Female, 20s	Afghanistan	October 19	October 19	No	18.18	Unknown
2	Male, 30s	Afghanistan	August 21	August 21	Unknown	21.08	No
3	Male 20s	Denmark	October 18	October 18	No	23.18	Unknown
4	Female, <10	Afghanistan	November 2	November 3	No	28.12	No
5	Female, 10-19	Germany	September 12	September 13	No	28.14	No
6	Male, 30s	India	Unknown	August 21	Unknown	29.12	Unknown
7	Female, 30s	Pakistan	November 2	November 3	Yes	29.67	Yes^a^
8	Male, 10-19	Afghanistan	August 26	August 27	No	31.36	No
9	Male, 30s	Belgium	November 18	September 19	No	31.90	Unknown
10	Female, 30s	Afghanistan	August 26	August 26	Unknown	34.41	Unknown
11	Female, <10	Afghanistan	Unknown	August 25	Yes	34.50	Unknown
12	Female, 60s	Austria	October 18	October 18	No	35.23	No
13	Male, 60s	Australia	November 25	November 26	No	35.41	No
14	Female, <10	Iran	October 19	October 20	No	35.65	No
15	Male, 10-19	Afghanistan	September 8	September 16^b^	No	37.37	No
16	Female, 30s	US	September 11	September 11	Yes	37.58	No

^a^ Individual received the Johnson & Johnson vaccine (date unknown).

^b^ This individual tested negative on September 8, 22, and 29, 2021.

## Discussion

As of March 19, 2022, Afghanistan had reported a total of 177 093 SARS-CoV-2 infections and 7654 deaths.^6^ With a population of approximately 40 million, Afghanistan had conducted 911 530 tests.^6^ With a strained and inadequate public health infrastructure, a high rate of infection transmission and a high incidence of infection would be expected. Whether the rugged mountainous geography of Afghanistan, which makes travel and intermingling difficult, its relative isolation from the rest of the world during the conflict years, or other unknown factors are reasons for these findings is unknown.

The low rate of infection among the evacuee population may be expected if a robust pretravel testing and screening program prevented infected persons from traveling. However, the evacuees in this cross-sectional study mostly left in a chaotic emergency situation with no known testing occurring prior to their leaving Afghanistan. A biased selection of those tested may also explain such findings. However, every person who entered Qatar from among the evacuees was tested. Limitations of this study include lack of accurate information on pretravel testing, and testing of only those who entered Qatar after evacuation. Information on the vaccination status was also not available for most evacuees.

The low rate of SARS-CoV-2 infection among evacuees from a major global armed conflict zone is reassuring to the concerned population, as well as countries receiving those evacuees. Whether these dynamics have changed in more recent weeks because of the global spread of the more infectious Omicron variant needs further investigation.
